# Probability-Based Algorithm for Bearing Diagnosis with Untrained Spall Sizes

**DOI:** 10.3390/s20051298

**Published:** 2020-02-27

**Authors:** Ido Tam, Meir Kalech, Lior Rokach, Eyal Madar, Jacob Bortman, Renata Klein

**Affiliations:** 1 Department of Software and Information Systems Engineering, Faculty of Engineering Sciences, Ben-Gurion University of the Negev, Beer-Sheva 85104, Israel; kalechm@gmail.com (M.K.); liorrk@bgu.ac.il (L.R.); 2 Pearlstone Center for Aeronautical Engineering Studies and Laboratory for Mechanical Health Monitoring, Department of Mechanical Engineering, Ben-Gurion University of the Negev, P.O. Box 653, Beer Sheva 8410501, Israel; eyalmad@post.bgu.ac.il (E.M.); jacbort@gmail.com (J.B.); 3 R.K. Diagnostics, P.O. Box 101, Gilon, D.N. Misgav 20103, Israel; Renata.Klein@rkdiagnostics.co.il

**Keywords:** machine learning, bearing diagnosis, hybrid model

## Abstract

Bearing spall detection and predicting its size are great challenges. Model-based simulation is a well-known traditional approach to physically model the influence of the spall on the bearing. Building a physical model is challenging due to the bearing complexity and the expert knowledge required to build such a model. Obviously, building a partial physical model for some of the spall sizes is easier. In this paper, we propose a machine-learning algorithm, called Probability-Based Forest, that uses a partial physical model. First, the behavior of some of the spall sizes is physically modeled and a simulator based on this model generates scenarios for these spall sizes in different conditions. Then, the machine-learning algorithm trains these scenarios to generate a prediction model of spall sizes even for those that have not been modeled by the physical model. Feature extraction is a key factor in the success of this approach. We extract features using two traditional approaches: statistical and physical, and an additional new approach: Time Series FeatuRe Extraction based on Scalable Hypothesis tests (TSFRESH). Experimental evaluation with well-known physical model shows that our approach achieves high accuracy, even in cases that have not been modeled by the physical model. Also, we show that the TSFRESH feature-extraction approach achieves the highest accuracy.

## 1. Introduction

Rotating systems in general, and specifically bearings, are essential elements in many mechanical systems such as vehicles, aircrafts, and industrial plants [1]. Failure in these systems can cause great economical loss or even risk of human life. For instance, in helicopters, the rotating systems are the main part of the swash-plate and the helicopter tail. A failure in one of them can lock out the whole helicopter from operation. In the last years, there is a demand to integrate intelligent algorithms in such systems to maintain rotating-system failures.

Model-based simulation is a well-known traditional approach to addressing this challenge [2]. The diagnostic process relies on an explicit model of the normal system behavior, its structure, and/or its known faults. Behavioral models depict the normal behavior of the system by a set of analytical equations or logical formulas. Inconsistencies between the observed behavior and the expected behavior produced by the model are suspected to be caused by faults. Fault isolation is the process of isolating the component(s) which explain these inconsistencies. Fault diagnosis is the process of identifying the mode of the fault. The mode of the fault is domain dependent. For instance, assume a model-based simulation of the influence of a spall on the bearings’ accelerations. Diagnosing a spall mode is the process of identifying the size of the spall given the accelerometer’s signals.

One drawback of this approach is the time consumption associated with building a model. Building such a model is challenging due to the bearing complexity and the expert knowledge required to build such a model. Obviously, building a partial physical model for some of the spall sizes is easier.

In the last years, some works integrate machine-learning (ML) algorithms together with the physical model to improve the diagnosis process. Integrating ML algorithms can improve the accuracy of bearing fault diagnosis. In this paper, we propose a machine-learning algorithm, called Probability-Based Forest, that uses a partial physical model. First, the behavior of some of the spall sizes is physically modeled and a simulator based on this model generates scenarios for these spall sizes in different conditions. Then, the ML algorithm trains these scenarios to generate a prediction model of spall sizes even for those that have not been modeled by the physical model.

Integrating ML techniques in this problem poses several challenges that are handled in this paper. First, training an ML algorithm on scenarios generated for some spall sizes and predicting spalls in a larger range of spall size are challenging. To this end, we propose a method to compute the confidence of the classification of a new instance for all spall sizes. A second challenge is related to the high number of features compared to the number of instances in the training set. To overcome this challenge, we propose a unique ML model based on Random Forest, named Probability-Based Forest. The third challenge is extracting significant features. We extract features using two traditional approaches:statistical [3] and physical [4], and an additional new approach: Time Series FeatuRe Extraction based on Scalable Hypothesis tests (TSFRESH) [5]. We explore each set of features separately and compare their performance.

Experiments show two significant contributions of this research. First, the proposed Probability-Based Forest algorithm achieves high accuracy, even in cases that have not been modeled by the physical model. Second, the new feature extraction approach, TSFRESH, achieves better results than the traditional statistical features and the unique physical features.

## 2. Related Work

Fault Detection and Diagnosis (FDD) approaches are typically divided into two categories: data-driven and model-based. The next literature review will survey the data-driven and the model-based approaches. Then, we present a new research direction that has been gaining momentum in recent years, which integrates data driven and model-based approaches.

### 2.1. Model-Based Approaches

Model-based approaches are totally dependent on a priori knowledge about the system. The diagnostic process relies on an explicit model of the normal system behavior, its structure, and/or its known faults. Currently, most of the model-based methods for spall size estimation are based on frequency and/or time domain analysis of the acceleration signal [6]. Jena and Panigrahi use a continuous wavelet transform on the vibration signal to locate the entry and exit of the bearing in and out of the defect for determination of the passage period of the ball [7]. A new dynamic model that considers the finite size of the ball in order to more accurately predict the event of the ball’s entrance into the defect is presented by Ahmadi et al. [8].

### 2.2. Data-Driven Approaches

Data-driven approaches rely on sampled data to extract useful information that enable fault detection and diagnosis. In the last decades, industrial and academic researches use data-driven methods to improve diagnosis and prognosis of bearings. Vakharia et al. [9] compare between a supervised machine-learning method (SVM) and an unsupervised machine-learning method (SOM) for fault diagnosis in ball bearings. In their work, an experimental test rig has been used and vibration responses for healthy bearings and bearings with faults were obtained. The results show that SVM is more accurate due to its better generalization capability.

Hyunseok et al. [10] propose a scheme to extract features from two different sensors for fault diagnosis bearing rotor systems. The scheme learns vibration images of correlated signals in an unsupervised manner.

### 2.3. Integrating Model-Based and Data Driven

In the last decade, some works integrated model-based and data-driven models. By using wisely machine learning methods together with model-based methods, we can get the best of the two worlds. Only a few works propose a hybrid approach for diagnosis of bearings. Goebel et al. [11] introduce an approach to fusing competing prediction algorithms for prognostics using two independent methods: physics-based model and experience-based model. The physics-based model estimates future operating conditions to determine future bearing conditions and returns a probability density function of the bearing remaining useful life [12].

Urko et al. [13] propose an architecture to implement hybrid modelling based on the fusion of real data and synthetic data. The proposed hybrid modelling architecture is validated in the field of rotating machinery, where the goal is to improve diagnosis and prognosis and, consequently, to optimize maintenance. A multi-body model and a semi-supervised learning algorithm are used to perform the hybrid modelling. The algorithm they propose synthesizes synthetic and real data. The synthetic data of which the classes are related to the different steps of diagnosis and prognosis is known and labeled. On the other hand, the real data is unlabeled. They accommodate both kinds of data in a learning process called semi-supervised classification. The good results of this work demonstrate the strength of a hybrid model and its potential contribution to condition-based maintenance [14].

### 2.4. Prognostics

Diagnosing the spall size enables the operator to decide if the bearing should be replaced. The decision should consider several parameters such as the cost of the bearing, its role in the system, etc. Furthermore, bearing spall size diagnosis can be part of bearing degradation prognosis, which is a great challenge. Many attempts have been investigated to address this challenge. Zhang et al. [15] integrate three main components including principal component analysis, hidden Markov model, and an adaptive stochastic fault prediction model. Lu et al. [16] propose a prognostic algorithm using the variable forgetting factor, recursive least-square, combined with an auto-regressive and moving average model. Li et al. [17] propose a prognostics methodology based on improved R/S statistic and Fractional Brownian Motion (FBM). Ayhan et al. [18] propose to run a bank of parallel-running remaining useful life predictors to mitigate the risk of getting unstable results in contrast to a single predictor.

To conclude, many attempts have been investigated to diagnose bearings with model-based and data-driven approaches. To the best of our knowledge, no previous work proposed to build an ML model based on scenarios generated by a physical model to address spall sizes that have not been modeled by the physical model.

## 3. Background and Problem Definition

**Figure 1:**
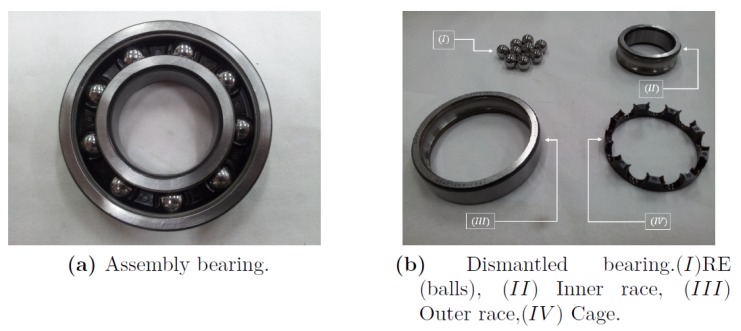
Ball bearing and its parts.

A rolling-element bearing is a key element in rotating machines. This term refers to various forms of bearings that use rolling of balls or rollers to reduce friction to a minimum, while enabling independent movement of two races. The bearing is considered as one unit, consisting of several parts: two steel rings, each of which has a hardened raceway; balls or rollers, also hardened, located between the two rings, and rolls on their raceway; and a cage, of which the role is to keep a fixed angular interval between the balls (Figure 1). The most common configuration of bearings allows rotation of a shaft relative to some fixed structure.

**Figure 2:**
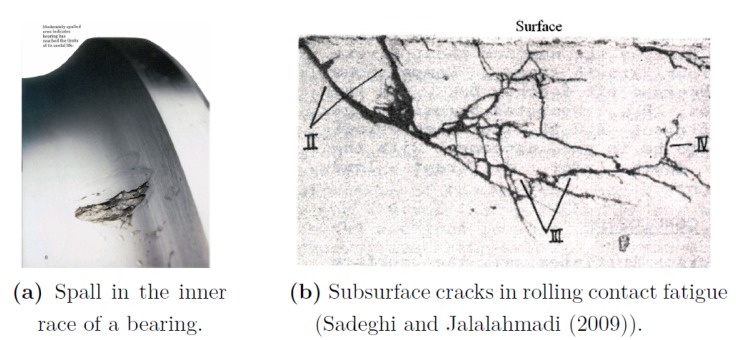
Spall from rolling contact fatigue.

A bearing may suffer from different failure modes such as corrosion, overheating, brinelling, contamination, etc. The most common failure mode is spalling [6]. Spalling is a process of surface deterioration in which a splinter or chip detaches and is removed from the surface of a larger body (Figure 2). In this paper, we focus on diagnosing spalls in the outer race of the bearing; that means determining the spall size. Within this research limitations, we assume that only **one** spall can exist in the outer race. We choose to focus on the outer race, but of course, the same process can be implemented for the inner race.

A well-known approach to coping with bearing defects is model-based diagnosis. A well-based physics-based model can simulate the system allowing examination of the kinematics and dynamics. Physical understanding of the processes occurring during the bearing rotations can improve the interpretation of measured signals from a defective bearing. Additionally, characterization of the physical phenomena in the measured signal contribute to choosing condition indicators which reflect the fault existence and severity [19].

In this research, we use a 3D general dynamic model for ball bearing. The model was developed in the PHM laboratory in Ben-Gurion University of the Negev [20]. The dynamic model allowed is based on the classical dynamic and kinematic equations. Several assumptions were used in the development of the model: The bearing parts act as rigid bodies which cause the local deformation to be neglected, the bearing races remain parallel to their initial position, and the angle between each rolling element is constrained by the bearing cage which neglects the bearing clearance. A local fault in one of the bearing races is implemented using the method presented in Reference [21]. Two circle elements which can be placed on the bearing outer or inner races represent the fault. The transmission function from the center of mass to a sensor is not considered. Using this method, we are able to characterize the physical phenomena that produce the signals measured in a real system. In this paper, we address the model as the “PHM model”.

The model allows monitoring of the dynamic and kinematics of each rigid body. From the simulations, some physical insights on the RE–spall interaction can be characterized. At the end of the process, we get the acceleration values for each axis. It is assumed that only one fault exists in the bearing static outer race.

The model outputs acceleration values in the bearing’s radial direction, i.e., the gravitational direction. In this paper, we call those values “synthetic raw data”. The raw data values change in each simulation depending on the state and the conditions of the bearing. These parameters could be controlled and obtained as input to the model. In particular, the parameters that can be controlled are the spall size, the rotating speed of the bearing (Rounds Per Second (RPS)), and the load.

As mentioned, the PHM model could simulate the acceleration values given the spall size, RPS, and load. However, diagnosis algorithms aim at the opposite function: to identify the size of the spall, given the outputted acceleration values. The PHM model was not planned to provide the opposite function. To cope with this challenge, we propose to use an ML approach. A ML model could determine the spall size given the acceleration values.

The classical process of machine-learning algorithms includes the following steps: First, measuring devices output signals that report the state of the system. Second, features are extracted from the signals’ raw data using various mathematical, statistical, physical, etc. methods. Third, an ML model with the relevant instances (training set) is trained and then tested with a test set.

ML methods for bearing diagnosis have been already proposed [22,23,24]. In this paper, we would like to go a step beyond in order to cope with a challenge of missing spall size instances in the training set. Generating a physical model is a well-known hard problem which requires experts to develop the model and to test their capabilities in private cases [19]. Usually, the model is not fully general for all spall sizes but for some of them. We propose an ML approach that trains a classification model with scenarios created by the physical model. The classification model will be trained to predict the spall size even in cases that have not been modeled by the physical model.

## 4. Learning a Model with Missing Spall Sizes

In this section, we describe a classification model that could predict the spall size even when the training data do not include samples of the whole spall sizes. Typically, a regression model could solve such problems; however, in the bearing diagnosis domain, engineers are not interested in predicting the exact spall size but a range of spall sizes. For instance, they are interested in knowing whether the spall size is in the range of [1 mm, 1.5 mm] rather than in knowing whether it is 1.2 mm or 1.4 mm. In this case, a classification model that predicts the spall size range is more suitable. To overcome the challenge of unseen spall sizes (classes) in the training set, we show a method to classify instances in case some of the classes have not been seen in the training set. To this end, we propose an equation to define the confidence of the trained and untrained classes. Our method proceeds as follows:

1. Record samples from the PHM model for the known spall sizes.
2. Extract features for these samples.
3. Train a classification model with these samples.
4. Extend the classification model for more spall sizes.

In our problem, we generate a training set by recording the acceleration values outputted by the PHM model for various input configurations. Each configuration defines an instance in the training set which includes the spall size, the rotating speed of the bearing (Rounds Per Second (RPS)), and the load. To classify the spall size, we use a multi-class classification method, where a class is the spall size. The ML classifier learns the mapping between the acceleration signature and the spall size for a certain load and RPS.

Next, we describe the feature extraction methods (Section 4.1), the classification model (Section 4.2), and the way we classify new instances (Section 4.3).

### 4.1. Feature Extraction

Feature extraction is a key factor in the success of an ML diagnosis. An instance in the training set includes the acceleration values of the bearing in three axes and its RPS and load values. We propose three approaches to extract features from the acceleration values: statistical, physical, and TSFRESH features. We explore each set of features separately and compare between their performance.

**Figure 3:**
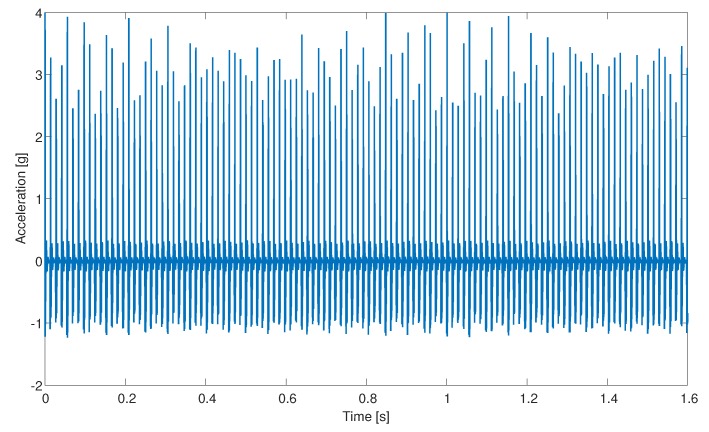
Simulated data from the PHM model in the time domain.

**Figure 4:**
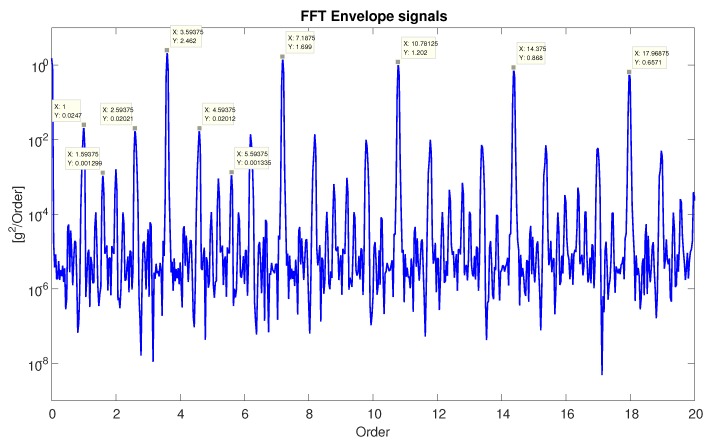
Simulated data from the PHM model in the order domain.

- **Statistical features:** We select 6 statistical features from the literature; here is a summary of the features proposed: mean value of the acceleration values, standard deviation, skewness, kurtosis, crest factor, and margin factor [9,23,25,26].
- **Physical features:** When a defected bearing spins, it produces a series of periodic impulses (Figure 3) that are usually caused by contact with the damaged surface on one of the bearing parts. The rate of these impulses is known as bearing tones. We use 1400 bearing-focused features, which are based on bearing tones and extracted from the order and envelope order domains [4]. An example of extracting the relevant bearing tones from the order domain is presented in Figure 4. The features are the bearing tone harmonics and their three sidebands (from both sides).
- **TSFRESH:** TSFRESH (Time Series FeatuRe Extraction based on Scalable Hypothesis tests) is a python package added in 2016 [5]. It automatically calculates many time series characteristics, the so-called features. This package includes hundreds of features from various fields. The features can be statistical equations like mean or skewness or can be features from different fields like absolute energy of the time series, Fourier coefficients of the one-dimensional discrete Fourier Transform for real input by fast, or the number of peaks of at least support *n* in a time series *x*.

Each set of the extracted features is fed to the ML models, but unlike a classical supervised learning process, we train with samples that include only labels of some of the spall sizes and test with the whole size.

### 4.2. Classification Model

We **initially** implement Random Forest since, in preliminary experiments, it has been proven as an efficient predictor. However, Random Forest poses a challenge in our research since there is a large number of features compared to the number of samples. Specifically, we have only a few hundreds or dozens of samples compared to thousands of physical and TSFRESH features. This can lead to overfitting and can make the ML model unreliable. To overcome this challenge, next, we present an alternative method to generate the decision trees in Random Forest.

#### Probability-Based Forest

In this section, we first describe the Random Forest algorithm and its drawbacks in handling large amounts of features compared to the small amount of samples. Next, we describe the Probability-Based Forest algorithm and its way of handling a large set of features.

The Random Forest (RF) algorithm generates an ensemble of multiple decision trees. RF selects randomly a subset of features to build each decision tree. Formally, let $TS=\{ts_{1},\ldots ,ts_{q}\}$ be a set of all samples in the training set. We define a feature as a pair of the feature name and its probability to be selected to generate a decision tree:

**Definition** **1** (Feature)**.**  *Feature f is a pair 〈L(f),P(f)〉, where L(f) stands for the label of the feature and P(f) stands for the probability of the feature to be selected to generate a decision tree. We denote $F=\{f_{i},\ldots ,f_{m}\}$ to represent the set of features of a sample in the training set TS and P(F) as the probability distribution of the features.*

In the RF algorithm, the distribution P(F) is uniform. Algorithm 1 describes the RF algorithm. In the main loop in line 2, the algorithm builds *n* decision trees. In each iteration, $F^{\prime }$ trees are selected from the set of features *F* according to the distribution of P(F) (uniform distribution). Those $F^{\prime }$ features are used to build the decision tree dt, which in turn added to the forest of decision trees DT.

Statistically, the larger and more diverse the set TS, the deeper the decision trees and the more features participate in building the tree. In our prediction problem (spall size prediction), there are much more features than instances in TS. Thus, it is expected that, among the selected features to generate decision tree dt, only a small number of features will actually build it. Moreover, when building a decision tree, the more the feature is significant, the higher it is located in the decision tree. In our case, since the features that actually build the trees are the most significant, we are expected to have approximately the same features in the whole decision trees, while other features have no opportunity to influence the decision trees.
**Algorithm 1:** Pseudo-code for Random Forest training stage.
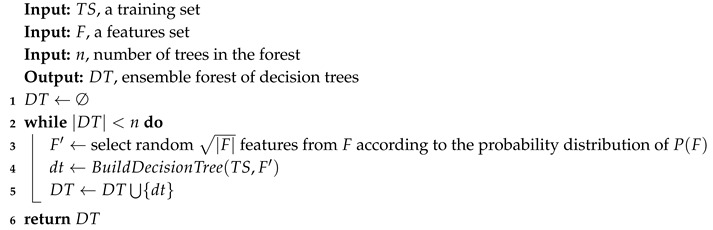


To overcome this drawback, we propose a Probability-Based Forest (PF). PF is also an ensemble of trees. The difference between the algorithms is the way the features are selected to generate the trees. While the distribution of P(F) in RF is union, in PF, P(F) changes with each iteration. We initialize P(F) with a uniform distribution. If a specific feature $f_{i}$ is selected and **actually participates** in building the decision tree, its probability to be selected again significantly reduces. This kind of forest allows more features to participate in constructing the trees.Furthermore, the features that construct the trees in Probability-Based Forest differ from one decision tree to another. This variation stems from the fact that we statistically prioritize insignificant features over previously selected significant features. When a feature is selected to generate a tree, its probability to be selected again reduces, which means that the probability of a less significant features increases.

When classifying a new instance with RF, the whole tree classifies the instance and the classification is determined by the majority. Applying this policy to PF is unfair since some of the trees are built from more significant features than others and, thus, their classification is expected to be more accurate. To this end, we define a weight for each decision tree in the forest.

**Definition** **2** (Decision tree)**.**  *A decision tree dt is a pair 〈L(dt),W(dt)〉, where L(f) stands for the label of the decision tree and W(dt) stands for the weight of the decision tree in the forest. $\sum_{i=1}^{n}W\left(dt_{i}\right)=1$.*

W(dt) is computed by the accuracy of the decision tree. The more accurate the tree, the higher its weight. The accuracy is computed by a cross-validation method. The PF algorithm divides the samples to five folds and, by a cross-validation method, calculates the average accuracy of each tree. Let Acc(dt) stand for the averaged accuracy of dt in the cross validation; then, $W\left(dt\right)=\frac{Acc(dt)}{\sum_{i=1}^{n}Acc\left(dt_{i}\right)}$.

Algorithm 2 presents the pseudo code of the PF algorithm. This algorithm is similar to Algorithm 1, but in the main loop in line 8, the probabilities of the features that have participated in the decision tree are decreased. Finally, at the end of the algorithm, the weights of the trees are calculated.

Beyond the classification of a new instance to one of the given classes, PF returns a confidence for each one of these classes. Let us define $C_{i,j}$ as the confidence of classifying a new instance to class $s_{i}$ based on decision tree $dt_{j}$.

The confidence values for *r* classes and *n* trees in the forest can be represented in a matrix view:

$$C=\left(\begin{array}{cccc} C_{1,1} & C_{1,2} & \ldots & C_{1,n}\\ C_{2,1} & C_{2,2} & \ldots & C_{2,n}\\ \vdots & \vdots & \ldots & C_{3,n}\\ C_{r,1} & C_{r,2} & \ldots & C_{r,n} \end{array}\right)$$

**Algorithm 2:** Pseudo-code for Probability-Based Forest training stage.
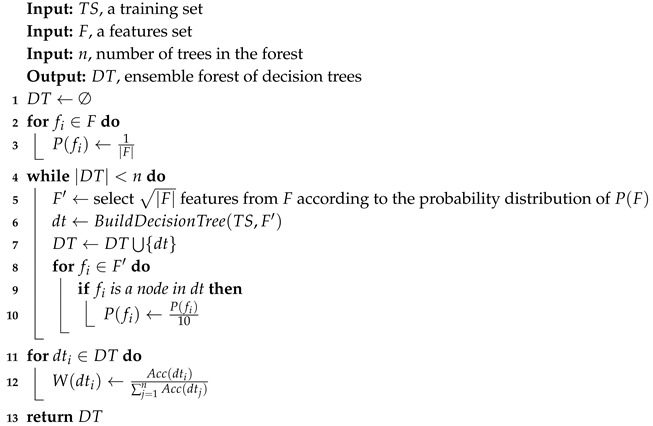


Recall $W(dt_{i})$ is the weight of decision tree $dt_{i}$; we can abbreviate it as $W_{i}$ and represent it in a vector view:

$$W=\left(\begin{array}{c} W_{1}\\ W_{2}\\ \vdots \\ W_{n} \end{array}\right)$$

Then, given a the confidence of the classes in the trees and the weights of the trees, their cross product returns the weighted average confidence of each class.

$$C_{PF}=\left(\begin{array}{cccc} C_{1,1} & C_{1,2} & \ldots & C_{1,n}\\ C_{2,1} & C_{2,2} & \ldots & C_{2,n}\\ \vdots & \vdots & \ldots & C_{3,n}\\ C_{r,1} & C_{r,2} & \ldots & C_{r,n} \end{array}\right)\times \left(\begin{array}{c} W_{1}\\ W_{2}\\ \vdots \\ W_{n} \end{array}\right)=\left(\begin{array}{c} C_{1}\\ C_{2}\\ \vdots \\ C_{r} \end{array}\right)$$

where $C_{i}$ is the confidence of PF by classifying the new instance to class $s_{i}$ given the whole trees’ classification.

For example, assume n=3, which means that DT contains three trees $DT=\{dt_{1},dt_{2},dt_{3}\}$ and that their appropriate accuracies are (0.9,0.7,0.6), respectively. Their normalized weights are W=(0.409,0.318,0.273), respectively. Assume the prediction confidence of the trees over a new instance is as follow:

$C_{1,1}=0.2,C_{2,1}=0.6,C_{3,1}=0.1,C_{4,1}=0.1$
$C_{1,2}=0.4,C_{2,2}=0.4,C_{3,2}=0.1,C_{4,2}=0.2$
$C_{1,3}=0.5,C_{2,3}=0.4,C_{3,3}=0.0,C_{4,3}=0.1$

Then, PF confidence is as follows:

$$C_{PF}=\left(\begin{array}{ccc} 0.2 & 0.3 & 0.5\\ 0.6 & 0.4 & 0.4\\ 0.1 & 0.1 & 0.0\\ 0.1 & 0.2 & 0.1 \end{array}\right)\times \left(\begin{array}{c} 0.409\\ 0.318\\ 0.273 \end{array}\right)=\left(\begin{array}{c} 0.314\\ 0.482\\ 0.073\\ 0.132 \end{array}\right)$$

### 4.3. Classifying Untrained Classes

To overcome the challenge of classifying instances with a spall size that has not been trained, in this section, we describe how our classifier determines the spall size of such instances. In addition, we present an equation to define the confidence of the trained and untrained spall sizes.

Let $T=\{s_{1},\ldots ,s_{a}\}$ be the set of classes (spall sizes) in which the classifier trained on $UT=\{s_{a+1},\ldots ,s_{b}\}$ is the set of classes that have not been trained by the classifier in the training phase. Given a new instance, a typical multi-class classifier computes a confidence for each one of the classes in set *T*. However, in our model, the classifier will not compute a confidence to classes that have not been in the training set (UT). Thus, we use the confidence given to the classes in set *T* in order to compute the confidence of each one of the classes in UT.

The computational method we propose for the confidence is appropriate for cases where the classes in the sets T∪UT can be linearly ordered. For instance, T={1,2.3,3.6,5}, UT={1.6,3.0,4.3}, and the linear order is {1,1.6,2.3,3.0,3.6,4.3,5}.

Our method includes two steps. First, we compute the predicted spall size by linear interpolation:

(1) $$predict=\sum_{i=1}^{a}s_{i}\cdot C_{i}$$

where, $s_{i}$ is the value of the class (spall size) and $C_{i}$ is the confidence of PF by classifying the new instance to class $s_{i}$ given the whole trees’ classification.

Then, we use this prediction to compute the confidence of each spall size. Given max=max(T∪UT) and min=min(T∪UT),

(2) $$c_{i}=\frac{(\left(max-min\right)-|predict-s_{i}{\left|\right)}^{x}}{\sum_{i=min}^{max}(\left(max-min\right)-|predict-s_{i}{\left|\right)}^{x}}$$

The closer the predicted spall size to a given spall size class ($predict-s_{i}$), the greater its confidence. The denominator is only used for normalization purpose. *x* is used to control the penalty given to those spall sizes that are far from the predicted spall size. The greater *x*, the greater the penalty.

For instance, continuing our previous example, assume the confidence of a new instance over *T* as follows: 〈1:0.314〉,〈2.3:0.482〉,〈3.6:0.073〉,〈5:0.132〉, where the pair 〈y:z〉 represents the spall size and its confidence by the classifier. By Equation (1), we get predict=1·0.314+2.3·0.482+3.6·0.073+5·0.132=2.343.

It is easy to see that the closest spall size to this prediction is 2.3 mm. Indeed, the confidence computed by Equation (2) to this class is the highest. For instance, for x=1, max=5, and min=1, the nominator value is 4−|2.343−2.3|=3.957. The normalized scores for the different spall sizes are

〈1:0.137〉,〈1.6:0.168〉,〈2.3:0.205〉,〈3:0.173〉,〈3.6:0.142〉,〈4.3:0.106〉,〈5:0.069〉

The normalized confidences when x=2 are

〈1:0.122〉,〈1.6:0.183〉,〈2.3:0.270〉,〈3:0.193〉,〈3.6:0.130〉,〈4.3:0.072〉,〈5:0.031〉

This example shows that increasing *x* reduces the confidence of the distant classes. Controlling *x* is important for the evaluation of the AUC as presented in Section 5.

### 4.4. Summary

The following summarizes the whole process of the suggested methodology. First, we generate acceleration value data by the PHM model for different spall sizes, RPS, and load values. Second, we extract statistical/physical/TSFRESH features. Third, we train a multi-class classification model using our Probability-Based Forest classification method. Then, given a new instance, we predict its spall size by the classification model using the confidence function we proposed.

## 5. Evaluation

In this section, we present experimental evaluation to examine the accuracy of our method and to test the impact of the feature set types on the accuracy. In particular, we would like to address the following research questions:

**RQ1.**  How accurate is the classification of untrained classes?
  RQ1 asks what is the accuracy of the multi-class classification model described above when trained on instances labeled by some classes and tested on instances including trained and untrained classes.
**RQ2.**  To what extent do the load and RPS influence the accuracy?
  RQ2 asks whether training with varied and noisy loads and RPS values influence the accuracy of the classification model.
**RQ3.**  Which set of features has the best accuracy?
  RQ3 asks which one of the feature sets (statistical/physical/TSFRESH) is the most significant for the accuracy of the classification.
**RQ4.**  Does the Probability-Based Forest algorithm improve the accuracy?
  RQ4 aims to evaluate our new algorithm (PF) that addresses the challenge of the small amount of samples compaed to large amounts of features.
**RQ5.**  What is the impact of the number of untrained classes on the accuracy?
  Obviously, the more untrained classes in the training set, the lower the accuracy. RQ5 asks to what extent this factor influences the accuracy.

### 5.1. Experimental Setup

To evaluate our method, we generate raw data by a physical model developed by the PHM lab in Ben-Gurion University [19]. We simulate different states of operation to get different raw data. In this section, we describe the raw data generation process and the performance metrics of our methods.

#### 5.1.1. Generate Raw Data

The synthetic raw data is actually the acceleration values on the three axes: radial, axial, and tangential, measured by the accelerometer placed on the outer race. To have a wide range of examples, we ran 2625 different states of operation. These states includes 7 spall sizes (1.0 mm, 1.6 mm, 2.3 mm, 3.0 mm, 3.6 mm, 4.3 mm, and 5.0 mm) (Note that, although the spall sizes are discrete, each spall size actually presents a range of spall sizes. For instance, 1.6 represents a range of [1.3 mm,1.9 mm].), 5 load sizes (10, 30, 50, 70, and 90), and 3 rotating speed (RPS) sizes (20, 40, and 60). To simulate closer real-world situations, we added for each of these load and RPS values four additional noisy operational states by adding a noise of ±5% and ±10%. Table 1 summarizes the parameters of the experiments.

To answer RQ1, we selected randomly 20% (525) samples from the full dataset to use as a test set. This includes data on 1–5 mm spall sizes. Then, we removed from the rest of the data the samples that have spall sizes of 1.6 mm, 3.0 mm, and 4.3 mm. These samples are used as the untrained samples. The rest of data is used for the training set. In that way, we could train a classifier with missing spall sizes in the training set and test on the whole spall size. We repeated this process of selecting random 20% of the instances 14 times. Thus, statistically, more than 95% of the data set samples are selected to participate in building the training model at least one time.

To answer RQ2, we generated additional four training sets to evaluate the impact of each one of the environmental parameters that influence the classification model. (1) Un Noisy RPS: this configuration keeps the RPS with no noise in the training set. Comparing the accuracy of the classification model when training with and without noisy RPS can indicate to what extent training with noisy RPS is important. (2) Un Noisy Load: this configuration keeps the load with no noise in the training set. (3) Constant RPS: this configuration keeps the training set with instances that includ only a fixed 20 RPS. (4) Constant Load: this configuration keeps the training set with instances that include only a fixed 10 load. Table 2 summarizes the number of samples used to learn each one of the models.

To answer RQ3, we run each one of the classifiers we generated for the different configurations, with each one of the feature sets: statistical, physical, and TSFRESH. To answer RQ4, we ran the whole process on both the Random Forest classifier and the Probability-Based Forest classifiers. Finally, to answer RQ5, we run the different configurations with additional untrained classes in the training set.

#### 5.1.2. Metrics

**Figure 5:**
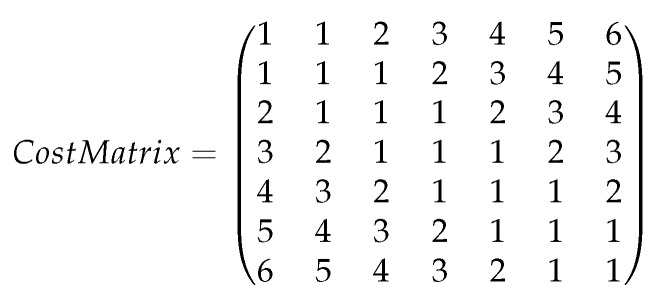
An example of a cost matrix.

We measure the classification models with standard machine-learning metrics: accuracy, precision, recall, F1-score, and AUC. Computing these evaluation metrics for multi-class should take into consideration not only whether the instance is classified to the correct class but also the distance of the classification from the real class. Specifically, in the bearing spall-size classification, when classifying an instance of spall size 3 mm, we may want to give a greater penalty for a classifier that classifies it to a spall size of 1 mm than a classifier that classifies it to 2.3 mm. The greater the difference between the real and predicted spall sizes, the greater the penalty. In Equation (3), we present the computation of this penalty, and in Figure 5, we present the cost matrix based on the penalty computation. The rows represent the predicted spall size, and the columns represent the real spall size. For instance, when classifying a spall size of 1 mm to 5 mm, the penalty is 6 (upper-right corner).

(3) $$Penalty=\frac{\left|(real\;spall\;size\;-predicted\;spall\;size)\right|}{real\;spall\;size}*100\%$$

**Figure 6:**
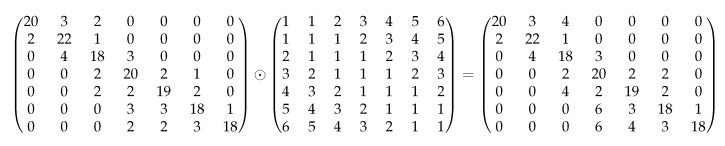
Example: the product of a confusion matrix by the cost matrix.

To consider the penalties in the classification output, we multiply the cost matrix with the Hadamard product by the classifier’s outputted confusion matrix. This product produces a cost-sensitive confusion matrix on which we perform the metrics’ calculation. For example, assume the outputted confusion matrix as presented on the left side of Figure 6. The accuracy of the classification model based on this confusing matrix is 0.771. After multiplying it by the cost matrix, we will get a cost-sensitive confusion matrix (on the right of Figure 6) with an accuracy result of 0.714.

Computing the AUC for a multi-class classification is more complicated, especially since we classify instances to classes that do not exist in the training set. For this, we compute the confidence of the classes as explained in Section 4, and based on it, we compute the AUC, as proposed by Reference [27]. This method computes an ROC curve for each one of the classes separately by considering the rest of the classes as one class and then returns the average AUC from all the generated ROC curves.

### 5.2. Results

In this section, we present the results addressing the research questions presented in the last section.

#### 5.2.1. Compare between Classifiers

**Figure 7:**
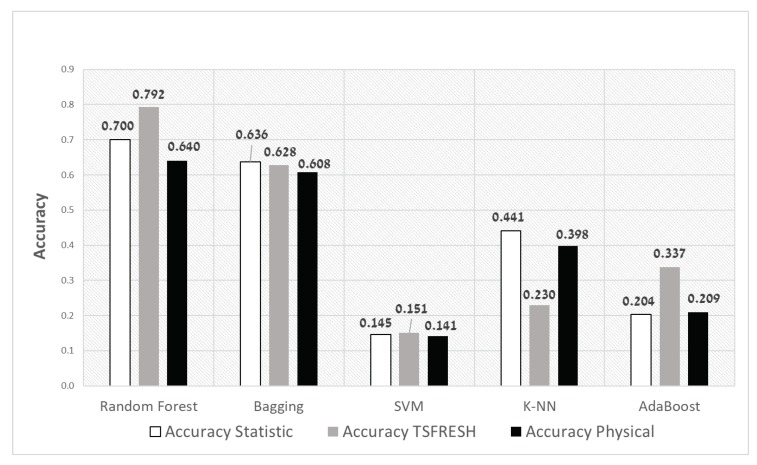
Compare accuracy between different classifiers with no penalty for different feature extraction methods.

As mentioned in Section 4.2, we initially implement the Random Forest classifier since, in preliminary experiments, it has been proven as an efficient predictor. In this section, we present experiments where we compare Random Forest classifier performance to other classifiers when draining with samples that include only labels of some of the spall sizes (T={1,2.3,3.6,5}), and testing with the whole size (T⋃UT={1.6,3.0,4.3}). We compare between five different classifiers: random forest, bagging, SVM, KNN (K nearest neighbors), and AdaBoost. Figure 7 presents the accuracy of the different classifiers for each one of the feature sets. It is clear that the accuracy of the Random Forest classifier is the highest compared to the other classifiers. In the rest of the experiments, we focus on the Random Forest performance.

#### 5.2.2. Predicting Unseen Spall Sizes

**Figure 8:**
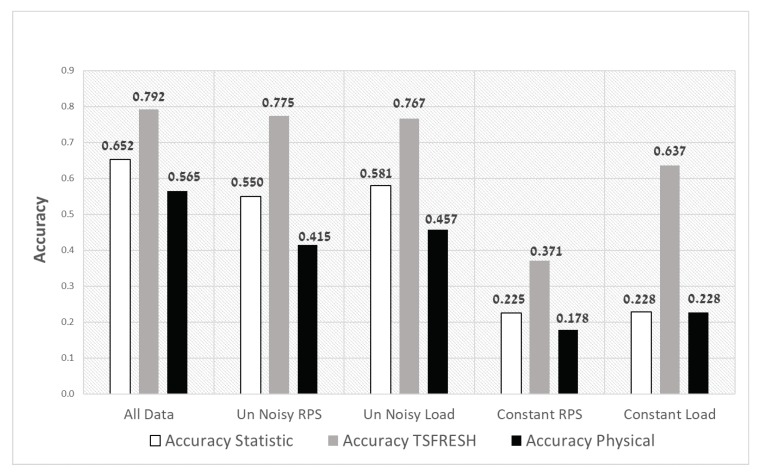
Accuracy of the Random Forest classifier for the different configurations and feature extraction methods.

Figure 8 presents the accuracy of the Random Forest classifier for five configurations using three features sets. The *x*-axis represents the configurations we tested (Table 2), where, for each configuration, we compare three feature sets.

The first contribution of our research **(RQ1)** can be observed in these results. The accuracy (0.792) of the all-data configuration is high. This proves that our ML method classifying untrained classes in the training set is useful.

A second observation can be seen by comparing the five configurations. It can be seen that the influence of noisy RPS and noisy load on the accuracy of the model is low. That means that, even without training with various noisy data, we can get almost the same accuracy as training with noisy data. This is not the case regarding the impact of the load and the RPS. The contribution of training with various RPS and especially with various load data is significant for the accuracy of the classifier. This finding is important for engineers that plan classification models for diagnosing bearing spall sizes, since the load and RPS should be considered as significant features. This observation addresses **RQ2**.

In addition, it is easy to see that TSFRESH has the highest accuracy for the whole configurations. This shows the third contribution of this paper **(RQ3)**. By extracting features with a novel method (TSFRESH) which does not need experts, we achieve better results than traditional feature extraction methods. Attempts to improve the accuracy by merging the three sets of features have not succeeded. The accuracy of the TSFRESH feature set alone gets better results than merging the three sets. The same trends can be seen in Table 3 for the other metrics: precision, recall, F1-score, and AUC. Appendix A presents the most significant features for bearing spall-size diagnosis as we conclude.

#### 5.2.3. The Impact of the Cost Matrix

**Figure 9:**
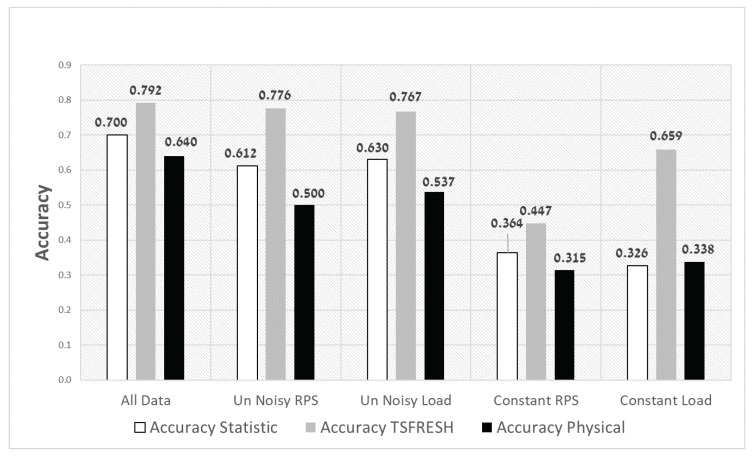
Accuracy of the Random Forest classifier with **no penalty** for different configurations and feature-extraction methods.

To further analyse the impact of the penalty given by the cost matrix (Figure 5), we ran the same experiments with no penalty. Figure 9 presents the accuracy for the different configurations. It can be seen that the accuracy of the classifier using the TSFRESH features remains almost the same as the results with penalty (Figure 8). The accuracy by using the statistical features is a little bit better compared to Figure 8, but the accuracy by using the physical features is significantly increased in the constant load configuration. In this configuration, the accuracy is even better than using the statistical features. A reasonable explanation is that the classifier with physical features has less false classifications than the classifier that uses statistical features. However, in case it classifies falsely, the error is high and so is the penalty.

#### 5.2.4. Probability-Based Forest Algorithm

**Figure 10:**
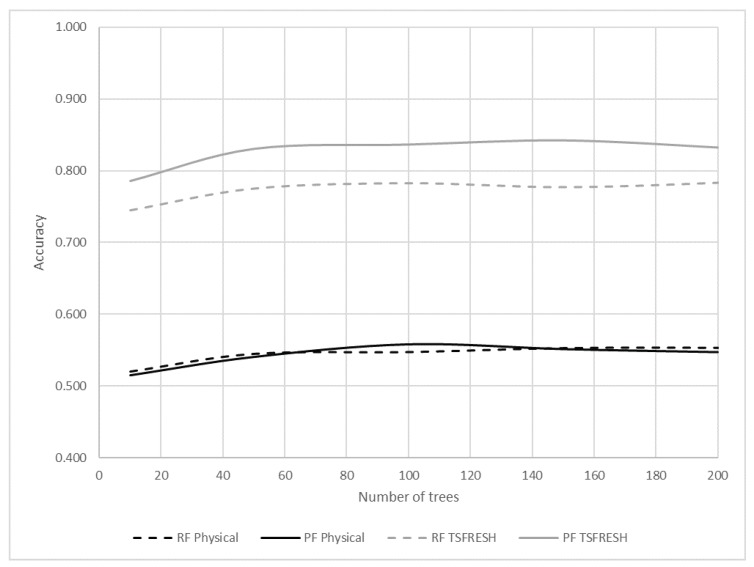
Accuracy of the classifiers when training on all data as a function of the number of trees in the forest-based classifiers.

In **RQ4**, we asked about the improvement of our Probability Forest algorithm. Figure 10 presents the accuracy of the classifiers when training on all data as a function of the number of trees in the Random Forest and the Probability-Based Forest classifiers. The dashed curves represent the Random Forest classifier and the solid curves represent the Probability-Based Forest classifier. The grey curves represent the TSFRESH feature set, and the black curves represent the physical set of features. It can be seen that the Probability-Based Forest algorithm using the TSFRESH feature set achieves the highest accuracy (0.842), significantly better than Random Forest (0.792). There is no significant difference between the algorithms when training with the physical features.

#### 5.2.5. The Impact of the Number of Untrained Spall Sizes

**Figure 11:**
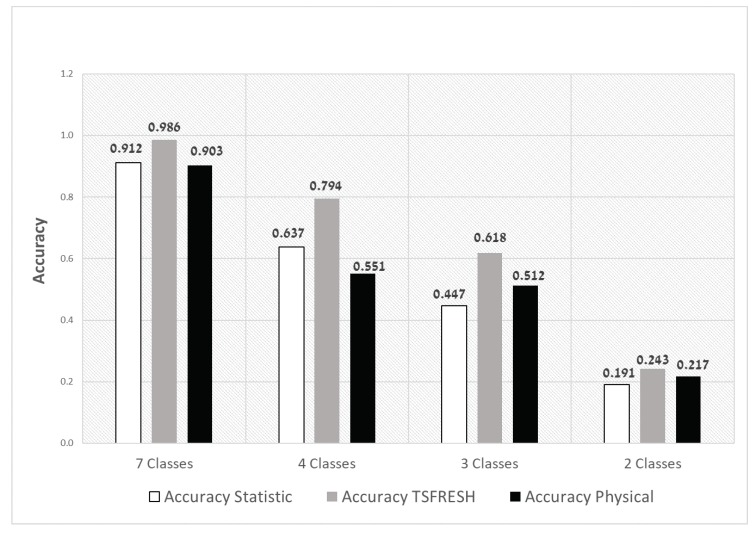
Accuracy of the Random Forest classifier for a different number of classes used for training.

In the experiments presented so far, we trained with four spall sizes (classes) and tested with seven spall sizes. In **RQ5**, we ask about the impact of the number of untrained spall sizes in the training set on the accuracy of the classifier. Figure 11 presents the accuracy of the Random Forest classifier for a different number of trained classes. The *x*-axis represents the configurations (the number of classes used for the RF training) we tested on, where, for each configuration, we compare the three feature sets. It is easy to see that TSFRESH has the highest accuracy for all the configurations. In addition, it can be seen that the higher the number of classes used for training, the higher the accuracy.

#### 5.2.6. Evaluation Conclusions

To conclude, we observe the the following:

1. The classification method we propose is helpful in predicting the spall size even for spall sizes that have been trained.
2. Training with noisy load and RPS is not as significant as training with various samples of load and RPS.
3. Using TSFRESH, feature extraction achieves the best results, better than using traditional feature-extraction methods.
4. Our Probability-based Forest algorithm improves the accuracy of the classification model compared to Random Forest.
5. The more untrained spall sizes in the training, set the less accurate the classification model.
6. The Random Forest classifier has the best performance compared to other classifiers.

## 6. Conclusions

In this research, we introduced a novel hybrid approach that integrates machine-learning techniques with a physical model to classify untrained spall sizes. Our approach proposes to use a physical model to produce simulated instances for the scenarios it planned to simulate. Then, a machine-learning technique is used to learn a classification model to address untrained scenarios. Evaluation with the PHM physical model and the Probability-Based Forest algorithm showed that this approach could achieve high accuracy even in untrained cases. In addition, we compared three sets of features and concluded that the TSFRESH feature extraction approach produces the highest accuracy. Finally, we presented the benefit of the Probability-Based Forest algorithm to overcome the challenge of large amounts of features compared to a few number of samples.

In future work, we plan to evaluate our method on real-data spalls. This is challenging since, beyond the natural difficulties with real-data, we should develop a transmission function from the center of mass of the bearing to the sensor. This is a great challenge. In addition, in this paper, the sets of *T* and UT are linearly ordered based on spall sizes. In future work, we plan to predict spall sizes out of the boundaries of the trained spall sizes. For instance, use a training set of spall sizes between 1 to 3.6 and predict unseen cases of 4.3 and 5. An extreme case of this scenario is to train with only normal data and to predict some spall sizes.

## Figures and Tables

**Table 1:** The experimented parameters for generating the instances using the physical model.

Spall Size	Load	RPS	Total
1.0 mm	10 ±5%,±10%	20 ±5%,±10%	
1.6 mm	30 ±5%,±10%	40 ±5%,±10%	
2.3 mm	50 ±5%,±10%	60 ±5%,±10%	
3.0 mm	70 ±5%,±10%		
3.6 mm	90 ±5%,±10%		
4.3 mm			
5.0 mm			
**7**	**25**	**15**	**2625**

**Table 2:** The experimented configurations and the size of the training set when using four spall sizes for training.

Configuration	#Training Set
All Data	1200
Un Noisy RPS	240
Un Noisy Load	240
Constant RPS = 20	400
Constant Load = 10	240

**Table 3:** Metrics summary of the classifier for different configurations and feature extraction methods.

Configuration	Features Set	Precision	Recall	F1-Score	AUC
All Data	Statistical	0.702	0.683	0.652	0.74
	TSFRESH	0.853	0.794	0.752	0.77
	Physical	0.645	0.606	0.568	0.72
Un Noisy RPS	Statistical	0.600	0.572	0.559	0.73
	TSFRESH	0.827	0.779	0.736	0.76
	Physical	0.541	0.441	0.425	0.69
Un Noisy Load	Statistical	0.625	0.594	0.583	0.74
	TSFRESH	0.822	0.766	0.723	0.76
	Physical	0.553	0.493	0.471	0.70
Constant RPS	Statistical	0.384	0.264	0.225	0.66
	TSFRESH	0.459	0.403	0.389	0.80
	Physical	0.482	0.246	0.193	0.64
Constant Load	Statistical	0.311	0.244	0.235	0.70
	TSFRESH	0.661	0.644	0.636	0.72
	Physical	0.449	0.285	0.236	0.65

**Figure A1:**
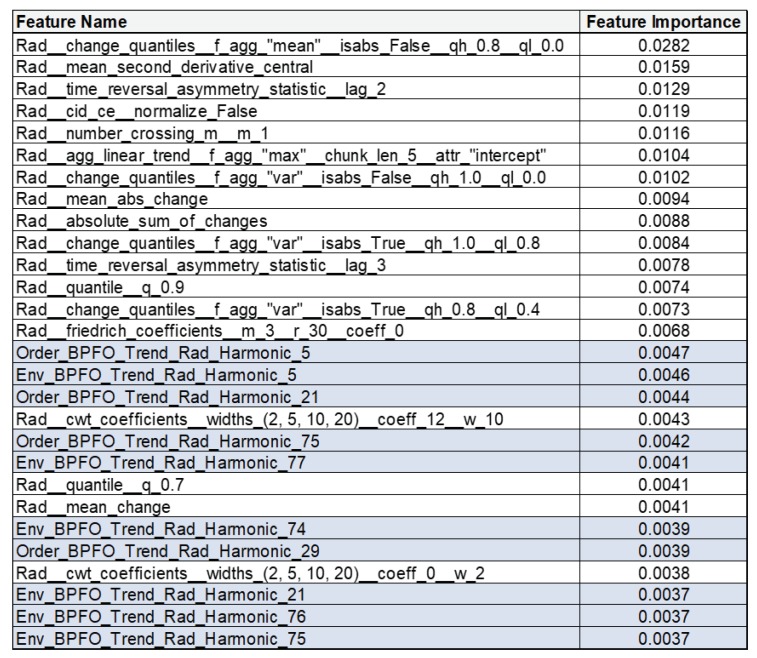
The most significant features for spall-size diagnosis.

## Appendix A

Figure A1 presents the 30 most significant features (ranked by their significance) for bearing spall-size diagnosis as concluded from the experiments using the Random Forest classifier. The features presented in Figure A1 are the outcome of the merged three feature sets (statistical, physical, and TSFRESH) used by the RF classifier. The left column represents the feature description, and the number in the right column represents its effect on the classification. The higher the number, the higher the effect. The features from the TSFRESH set are marked with white, and the features from the physical set are marked with light blue. The full description of the features from the TSFRESH set can be extracted from the web https://tsfresh.readthedocs.io/en/latest/text/list_of_features.html. To demonstrate it, let us focus on the second feature in the list. It returns the mean value of a central approximation of the second derivative. It is calculated by Equation (A1).

(A1) $$mean\;second\;derivative\;central=\frac{1}{n}\sum_{i=1}^{n-1}\frac{1}{2}\left(x_{i+2}-2\cdot x_{i+1}+x_{i}\right)$$

The physical features are calculated based on the classical bearing tones, i.e., BPFO (Ball Pass Frequency Outer race) [4] as we mentioned in Section 4.1.
